# Quantitative Evaluation of Performance in Interventional Neuroradiology: An Integrated Curriculum Featuring Theoretical and Practical Challenges

**DOI:** 10.1371/journal.pone.0148694

**Published:** 2016-02-05

**Authors:** Marielle Ernst, Levente Kriston, Javier M. Romero, Andreas M. Frölich, Olav Jansen, Jens Fiehler, Jan-Hendrik Buhk

**Affiliations:** 1 Center for Radiology and Endoscopy, Department of Diagnostic and Interventional Neuroradiology, University Medical Center Hamburg-Eppendorf, Martinistr. 52, 20246, Hamburg, Germany; 2 Department of Medical Psychology, University Medical Center Hamburg-Eppendorf, Martinistr. 52, 20246, Hamburg, Germany; 3 Department of Radiology, Division of Neuroradiology, Massachusetts General Hospital, Harvard Medical School, Boston, MA, United States of America; 4 Department of Radiology and Neuroradiology, University of Kiel, Arnold Heller Str. 3, 24105, Kiel, Germany; Heinrich-Heine University, GERMANY

## Abstract

**Purpose:**

We sought to develop a standardized curriculum capable of assessing key competencies in Interventional Neuroradiology by the use of models and simulators in an objective, quantitative, and efficient way. In this evaluation we analyzed the associations between the practical experience, theoretical knowledge, and the skills lab performance of interventionalists.

**Materials and Methods:**

We evaluated the endovascular skills of 26 participants of the Advanced Course in Endovascular Interventional Neuroradiology of the European Society of Neuroradiology with a set of three tasks (aneurysm coiling and thrombectomy in a virtual simulator and placement of an intra-aneurysmal flow disruptor in a flow model). Practical experience was assessed by a survey. Participants completed a written and oral examination to evaluate theoretical knowledge. Bivariate and multivariate analyses were performed.

**Results:**

In multivariate analysis knowledge of materials and techniques in Interventional Neuroradiology was moderately associated with skills in aneurysm coiling and thrombectomy. Experience in mechanical thrombectomy was moderately associated with thrombectomy skills, while age was negatively associated with thrombectomy skills. We found no significant association between age, sex, or work experience and skills in aneurysm coiling.

**Conclusion:**

Our study gives an example of how an integrated curriculum for reasonable and cost-effective assessment of key competences of an interventional neuroradiologist could look. In addition to traditional assessment of theoretical knowledge practical skills are measured by the use of endovascular simulators yielding objective, quantitative, and constructive data for the evaluation of the current performance status of participants as well as the evolution of their technical competency over time.

## Introduction

Given the increasing public interest in physician qualifications, several programs such as Continuous Professional Development (CPD), Maintenance of Certification (MOC), and Practice Quality Improvement (PQI) have been implemented during the last decade for ensuring high standards of medical practice. These structured assessment programs aim to stop the decline over time in a physician’s knowledge and skills as well as to improve care, to continuously reduce risk, to increase patients’ safety, and to provide better cost-benefit ratios.[1–3] While the aims and concepts of these programs are widely supported, the current approaches to achieve these goals are still a matter of debate. [2–4] In a highly specialized discipline like Interventional Neuroradiology (INR), the assessment of key competencies and procedural skills is challenging. Those opposed to the MOC program in its current form doubt that the performance on a written examination suffices as an adequate surrogate marker for overall competence in INR.[3] The ongoing development of endovascular procedural simulation applying either virtual reality (VR) or silicone vessel reproductions currently allows assessing reproducible measures of human performance in neuroendovascular procedures in an objective, quantitative, and constructive way.[5–9] Exploiting these possibilities of simulators, we sought to develop an integrated curriculum in order a) to assess key INR competencies and b) to analyze the associations between the practical experience, theoretical knowledge, and the skills lab performance of interventionalists. We hypothesized that practical skills are positively correlated with practical experience but not with theoretical knowledge.

## Methods

### Setting

All 26 participants of the Advanced Course in Endovascular Interventional Neuroradiology of the European Society of Neuroradiology held in Hamburg from January 26th to January 29th 2015 were invited and agreed to participate in the study. The study has been approved by the local ethics committee (Ethik-Kommission Ärztekammer Hamburg (WF-030/14) and the requirement for written informed consent was waived. Participants records and information was anonymized and de-identified prior to analysis.

### Assessment of experience in interventional neuroradiology

Prior to assessment, participants were invited to complete an online survey to rate their experience in interventional neuroradiology. The survey consisted of 10 questions regarding their current qualification as well as the number of procedures they assisted or performed as primary operator in aneurysm embolization, mechanical thrombectomy, and endovascular treatment of arteriovenous malformation (AVM) or dural Arteriovenous Fistula (dAVF). It was a multiple choice questionnaire with the opportunity to provide commentary for each question. In addition, data on demographic characteristics (sex, age) were collected.

### Assessment of participant performance skills

The course was set up similar to an Objective Structured Clinical Examination (OSCE). All participants had to fulfill specific tasks at different stations including VR simulators as well as a silicone vascular flow model set up in a dedicated neurointerventional catheter lab (Allura Clarity FD 20, Philips Healthcare, Best, The Netherlands).

On the VR simulator, participants were asked to perform a thrombectomy of a left sided M1 occlusion and to embolize an aneurysm of the Posterior Communicating Artery (PCOM) Region of the Internal Carotid Artery (ICA) (ANGIO Mentor, Simbionix, Cleveland, Ohio, USA). In the simulation software, the participants were free to select guidewire, diagnostic catheter, guiding catheter by type and diameter (all standard sizes were available). Prior to testing, all participants received a standardized didactic introduction. To minimize bias, there were no further instructions provided to any of the participants during the procedure. Procedure time, fluoroscopy time, amount of contrast agent, complications (e.g. wire perforation), correct first coil selection, and packing density were automatically assessed by the simulators and recorded for evaluation.

The silicone vascular flow model (Replicator, Vascular Simulations, Stony Brook NY) was a type of mechanical flow model simulating the cardiac cycle with a functional left atrium and ventricle. To duplicate the friction coefficients of catheters and endovascular devices the flow model was filled with a fluid with the viscosity of human blood. In the flow model participants had to embolize a three-dimensional reproduction of an Medial Cerebral Artery (MCA) aneurysm using an intrasaccular flow disrupter (WEB™ Aneurysm Embolization System, Sequent, California) under fluoroscopy. Total procedure time and fluoroscopy time were recorded. Correct positioning of the WEB-Device was assessed independently by two experts (M.E. and J.B.) using the published 3-grade WEB Occlusion Scale.[10] Readers were blind to the other observer’s assessments. Cases leading to a disagreement between the observers were reviewed by both readers to reach a consensus.

### Assessment of theoretical knowledge

At the end of the course, the participants had to complete an exam consisting of three parts. The first part included 20 multiple choice questions concerning neuroanatomy and neuroembryology, pathophysiology, material and techniques, as well as studies in INR. In the second part, participants had to answer 12 questions regarding treatment of a somewhat complex incidental aneurysm of the PCOM region of the ICA applying movies derived from live fluoroscopy of the real procedure to simulate a live case scenario. The third part consisted of a semi-structured standardized oral examination with questions covering the field of knowledge mentioned above supplemented by situational perceptivity as well as assessment and management of complications applying standardized case materials.

### Scoring

Experience, knowledge and skill domain scores were built as weighted composite scores from multiple components assed as surrogate measures of performance (see S1 Table). First, missing values were estimated from all available information using expectation-maximization. Second, as the components were captured by variously scaled measurements, all component scores were standardized to have a mean of zero and a standard deviation of one. Third, the weighted composite scores for each domain were calculated. With regard to the assessment of practical skills in aneurysm coiling, the packing density was essential and weighted the highest, followed by the time to final guide catheter position and the occurrence of complications such as perforation. Fluoroscopy time and a correct first coil selection were of secondary importance. Amount of contrast medium and the number of coils used were of minor importance. Concerning the evaluation of skills in thrombectomy, a short time to final guide catheter position was judged to be the most important factor. A short fluoroscopy time was favorable, while a small amount of contrast medium was considered relatively less important. With regard to the assessment of performance in aneurysm treatment with the WEB device, the correct placement of the WEB device was judged more important than the procedure time. After building the weighted sums, all domain scores were standardized again in order to allow for comparisons between domains.

### Statistical analyses

In bivariate and multivariate analyses, we investigated whether demographic (sex, age), experience (years, aneurysm coiling, thrombectomy, Flow-Disrupting devices), and knowledge (anatomy, materials and techniques in INR, studies, treatment) variables predict skills (aneurysm coiling, thrombectomy, WEB placement). First, we calculated pairwise Pearson product-moment correlations between each predictor and skill domain. Second, a linear regression model was fit including all predictors. Third, a stepwise linear regression model was fit with backward exclusion of all non-significant predictors. We considered associations with p < .05 as statistically significant but mark also results with p < .10 as statistical trends. All analyses were performed in R 3.1.0 (R Foundation for Statistical Computing, Vienna) and SPSS 21 (IBM, Armonk, NY).

## Results

### Participant characteristics and experience in interventional neuroradiology

All 26 respondents answered all questions in the survey, resulting in a response rate of 100%. Seven of the 26 participants were female (26.9%), and 20 participants were younger than 40 years (76.9%). 23 participants (88.5%) completed their radiology residency, one participant (3.8%) was in his fifth year of Radiology training, one (3.8%) in his first year (3.8%), and one participant (3.8%) was neurosurgeon. Each participant had at least one year of experience working in INR, with 12 (46.2%) reporting at least four years. The number of INR procedures performed by the participants is shown in Table 1. 10 of the 26 participants (38.5%) were certified neuroradiologists in their country, of whom three did not perform the minimum numbers of INR procedures as principle operator recommended by the Union of European Medical Specialists (UEMS) for acquiring competence in Endovascular Interventional Neuroradiology.[11]

**Table 1:** Participants’ experience in Interventional Neuroradiology.

	Number of interventions					
	none	n <5	n = 5–10	n = 11–50	n = 51–100	n >100
Intracranial aneurysms treated as first operator	4 (15.4%)	3 (11.5%)	5 (19.2%)	8 (30.8%)	3 (11.5%)	3 (11.5%)
Assisted intracranial aneurysms procedures	0	0	1 (3.8%)	9 (34.6%)	9 (34.6%)	7 (26.9%)
Thrombectomies performed as first operator	4 (15.4%)	7 (26.9%)	3 (11.5%)	7 (26.9%)	4 (15.4%)	1 (3.8%)
Assisted thrombectomies	2 (7.7%)	3 (11.5%)	3 (11.5%)	10 (38.5%)	5 (19.2%)	3 (11.5%)
Assisted endovascular treatments of pAVM or dAVF	0	3 (11.5%)	2 (7.7%)	17 (65.4%)	4 (15.4%)	0
Attended Flow-Disrupting Device procedures	9 (34.6%)	11 (42.3%)	2 (7.7%)	4 (15.4%)	0	0
Attended intracranial stenting procedures	2 (7.7%)	8 (30.8%)	5 (19.2%)	9 (34.6%)	2 (7.7%)	0

Note.—Data are the number of participants

### Knowledge

One participant did not attend the final examination. Only 5 participants answered at least 80% of the 20 multiple choice questions correctly (median 13/65% correct answers). The largest number of wrong answers occurred in the category ‘anatomy and embryology’. In the second ‘live case’ section of the examination only two participants answered less than 80% of the 12 questions correctly. In the final oral examination, 12 participants answered at least 80% of the 21 questions correctly and 6 participants answered less than 60%.

### Practical skills

Median procedure time, fluoroscopy time, time to final guide catheter position, amount of contrast medium and number of complications during thrombectomy of a left sided M1-occlusion and coiling of a PCOM aneurysm are shown in Table 2. Correct first coil selection was achieved 15 times, in median 8 coils were used (minimum 5, maximum 11). Concerning the embolization of an aneurysm-model of the MCA bifurcation using the WEB-Device, median procedure time was 27 minutes (minimum 9 min, maximum 32 min).

**Table 2:** Participants’ endovascular performance on a Virtual Reality Simulator.

	Median procedure time	Median fluoroscopy time	Median time to final guide catheter position	Median amount of contrast medium	Complications
**Thrombectomy of M1-occlusion**	09:52 min (05:37–31:30)	05:11 min (02:29–18:14)	04:31 min (00:17–09:56)	53 ml (11–330 ml)	none
**Aneurysm coiling**	23:29 min (14:02–49:53)	13:33 min (05:51–31:13)	06:31 min (04:25–11:56)	80 ml (26–236 ml)	n = 12

Note.—Data in parentheses are minimum and maximum.

### Associations between experience, knowledge, and skills

**Fig 1:**
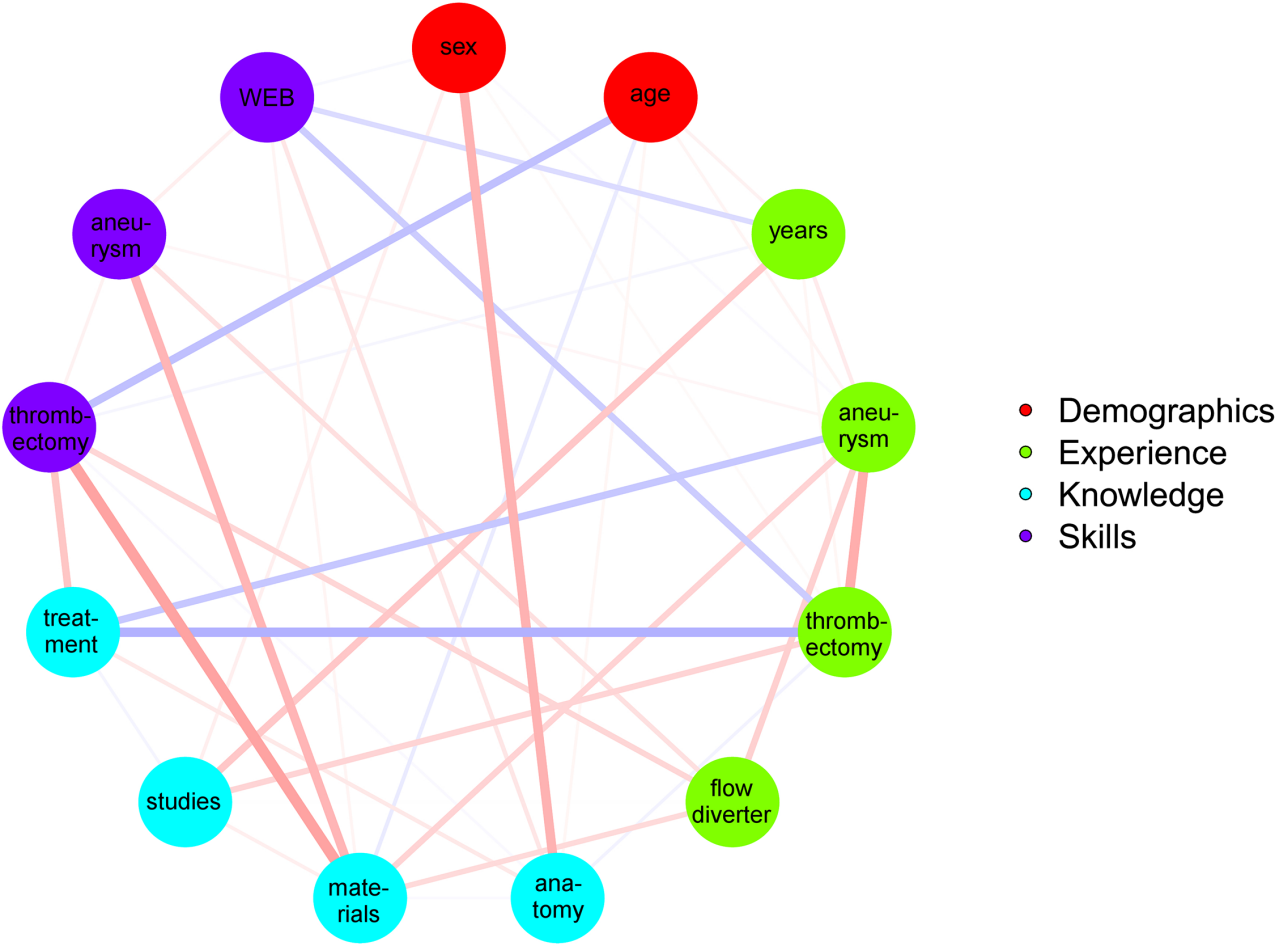
Associations between experience, knowledge, and skills.

Results of the bivariate and multivariate prediction of skills from experience and knowledge are shown in S2 Table and Fig 1.

Knowledge of materials and techniques was moderately associated with skills in aneurysm coiling (B = 0.431 [95% confidence interval 0.050 to 0.811]). In pairwise correlation analysis, knowledge of materials was strongly associated with thrombectomy skills (r = 0.491 [0.124 to 0.858]). There was a positive tendency concerning the association between knowledge of materials and performance in WEB treatment in regression with backward variable selection (B = 0.314 [-0.054 to 0.682]). A higher age was associated with lower thrombectomy skills (B = -0.356 [-0.664 to -0.049]). Experience in mechanical thrombectomy was moderately associated with thrombectomy skills (B = 0.396 [0.057 to 0.735]). We found a strong association between knowledge of treatment and thrombectomy skills (B = 0.562 [0.229 to 0.895]). Knowledge of anatomy was strongly associated with WEB treatment skills (B = 0.501 [0.101 to 0.900]). There was a slight tendency for female and more experienced participants to show lower skills in aneurysm treatment with the WEB device (B = -0.359 [-0.758 to 0.040] and -0.320 [-0.679 to 0.040], respectively). We found no significant association between age, sex, or work experience and skills in aneurysm coiling. Knowledge of studies was unrelated to practical skills.

**Fig 2:**
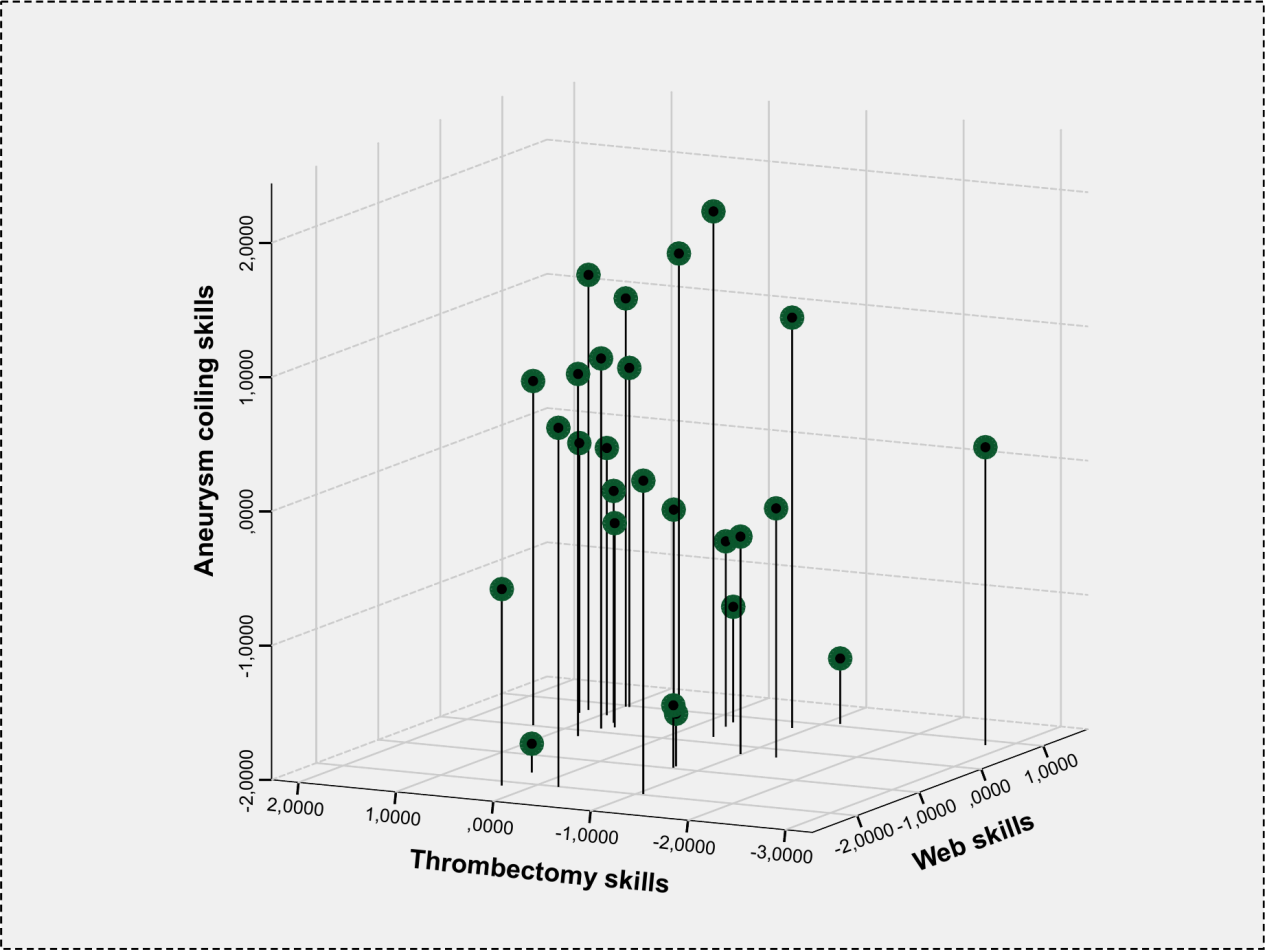
Relation between performance in mechanical thrombectomy, aneurysm coiling, and WEB placement.

The relation between performance in mechanical thrombectomy, aneurysm coiling and WEB placement is shown in Fig 2. Although none of these pairwise correlations was significant, there was a group of participants with very high skills level in all procedures.

## Discussion

Our study gives an example of a curriculum that reasonably and cost-effectively assesses the key competences of an interventional neuroradiologist in terms of theoretical knowledge and practical skills. This approach may help encountering the increased concerns and expectations regarding the monitoring and assurance of quality and safety in health care by offering objective test instruments, which can easily get adapted to new technologies and materials in a rapidly developing discipline like INR. Time of work experience does not guarantee wisdom and expertise. It was observed that the longer a physician is in practice, the poorer his clinical knowledge, judgment, and skills can become.[12] Similarly, we observed in our study that age is negatively associated with thrombectomy skills. Moreover, the number of treated patients with stroke or aneurysms might be a surrogate marker of work experience and good performance; however, the plain number of cases does not reveal how many procedures were successful und how many complications occurred. It is important to differentiate if the procedure was performed as primary operator or not, which therefore in our analysis was weighted higher than the number of procedures as assistant. Against our hypothesis, we observed significant associations between theoretical knowledge and practical skills (see Fig 1), e.g. between knowledge of anatomy and WEB treatment skills. Moreover, knowledge of material and techniques was associated with skills in aneurysm coiling and thrombectomy. Although our sample size was limited, it still fulfilled the recently suggested criterion of at least two subjects per variable in multivariate regression.[13] However, since the power of our analyses was rather low, associations had to be at least of moderate strength to be able to get detected at all. Concerning the relation between performance in mechanical thrombectomy, aneurysm coiling and WEB placement none of these pairwise correlations was significant in our study. However, compared to our daily experience, we do assume that there should be a strong correlation between the different skills and it is more likely that we simply could not proof it due to the low number of participants. Instead of claiming generalizability and proof of true correlations or even causal relationships, our correlation analysis should rather serve as a first example revealing the educational and scientific possibilities of a standardized quantitative performance assessment if performed in a larger cohort. To assess the theoretical knowledge of the participants, we created a multiple choice examination, as it is one of the most applied forms of standardized tests. According to UEMS recommendations, the questions covered a diversity of topics in neuroradiology from basic and clinical neurosciences to management of complications and research in INR.[11] However, multiple choice questions can be easier than open-ended questions, because it is easier to recognize an answer than to recall it. But it is less recognition we want to test for than the ability to synthesize and evaluate information or apply knowledge to solve complex problems.

In addition to the written examination, we performed an oral examination. To overcome the inconsistency of oral examinations, we had predefined areas of competence and traits to be assessed and thus designed standardized case materials. The results of the written multiple choice exam were weighted higher than the results of the oral examination in the statistical analyses, since these are more strongly standardized. Additionally, oral examination is excessively costly in terms of professional time and therefore cannot easily be recommended for a general certification process in INR. All in all, we believe that the performance on a written or oral examination does not suffice as adequate surrogate marker for overall competence in INR.

The assessment of procedural skills by the use of endovascular simulators is well recognized and has proved to be a feasible approach to yield objective data for evaluating technical competency.[5–9] Studies indicate that simulation not only improves learning but is especially effective in developing procedural skills.[14]

Currently two generally different approaches in modelling endovascular procedures exist on the market: computerized VR simulators and silicone vascular models, usually combined with a circulation pump. VR simulators are capable of recording the operator’s physical manipulation with the material and translating it into a digital performance assessment allowing quantitative evaluation. VR simulators have several other advantages: they do not require radiation dose and they are portable and customizable on a case by case basis. The simulator employed in this study was investigated in a recent study and rated favorably for both visual display and mechanical/haptic feedback properties by experienced fellows for the training of residents in diagnostic cerebral angiography.[7] In another study analyzing the realism, criterion validity, and training capability of VR simulators the ANGIO Mentor was judged useful as a teaching and training tool providing a realistic simulation of diagnostic cerebral angiography (face validity). Moreover the simulator was able to differentiate between individuals processing different levels of neurointerventional expertise level (criterion validity).[15] In terms of transferability of skills in the clinical setting, endovascular simulators should correlate to the participant’s competency in the angiosuite to provide a good evaluation of performance, notably if they become an assessment tool for certification. The study mentioned above also observed that inexperienced residents performed actions that were perceived as potentially dangerous while fellows with considerable experience in angiography performed the procedure with superior technique.[7] This might reflect a transfer of mechanical skills acquired in real-life cerebral angiography experience to the simulator. In reverse it strongly suggests that skills acquired on the simulator might be transferable to the real patients in the angiography suite. However, skills such as material preparation outside the patient cannot be trained and assessed by VR simulators. Further research is needed to validate the transferability of skills acquired on the simulator in the clinical setting and if simulator training finally improves safety and quality of patient care.

On the silicone vascular flow model simulating the cardiac cycle, the trainee is able to use the material in its dedicated “wet” environment allowing him to learn every step of a procedure. The replicator can be filled with a clear blood analog with a viscosity of human blood, thus duplicating the friction coefficients of endovascular devices, wires, and catheters as they traverse the vascular system. Another major advantage of the silicone model is that it can be used under realistic conditions in a catheter lab where the controls of the angiography unit and the effectiveness of using cones and collimators can be trained and assessed. The same silicone vascular flow model is used as a physician training tool for the WEB Intrasaccular Therapy Study (WEB-IT). One of the significant challenges of this study is to prove the safe and effective use of a new device by an entirely new user group (US clinical investigators) without roll-in cases for US FDA approval. Arthur et al. observed as benefits of using the Replicator a shortened learning curve as well as the practice of advanced techniques and more difficult delivery options in a high fidelity environment without compromising the patient’s safety. [16]

The current generation of VR simulators allows assessing many more parameters, such as haptic parameters, fine-motor digital movements, or motor and visual/spatial coordination. Simulation of complication is currently limited, but could in future serve as an important surrogate for cognitive ability to deal with the unexpected. However, in terms of cost-effectiveness, we decided to concentrate on established indicators for performance in aneurysm treatment and thrombectomy such as total procedure and fluoroscopy time and amount of contrast given during the intervention. This summative assessment was also necessary with regard to statistical analysis as the number of participants forced us to summarize variables. It is desirable that future simulators assess both more procedure-specific items and basic skills such as control over microcatheter movement. Further studies including a higher number of participants would allow a more detailed correlation analysis. We built weighted composite scores from multiple components as surrogate measures. For example, in aneurysm coiling the packing density was judged more important than the amount of contrast medium used. However, this is just a preliminary approach based on current practice and further research has to examine the validity of these surrogate measures of performance and might make an adaption of the weighting factors necessary.

All in all, we judge the quantitative measurement of performance applying a setup of variably simulated INR procedures a valuable supplemental tool in the assessment of performance in interventional neuroradiology. For sure, the use of endovascular simulators includes a large time demand on a limited number of instructors, increased work for all involved in the evaluation process and relatively high costs. Once installed, all procedures in the standardized test setup could be easily reproduced and adapted to different case scenarios for the coming generation of trainees. Additionally, it might give a good return to invest in the improvement as well as maintenance of performance of a neurointerventionalist. Thus a study analyzing the cost saving benefits of interventional stroke service observed that mechanical thrombectomy leads to major cost savings to the healthcare economy by improving clinical outcome and reducing the length of hospital stay of stroke patients.[17] Finally, further studies should investigate if the assessment of practical skills with the use of an endovascular simulator improves safety and quality of care.

The subdivision of overall performance in different domains such as experience, knowledge, and several practical skills and their quantitative assessment allows a visualization of the position of each individual in comparison to other participants (see Fig 2). Measuring the performance at several time points would allow following each participant over time, serving as orientation and feedback for improvement.

## Conclusion

Our study gives an example how an integrated curriculum for reasonable and cost-effective assessment of key competences of an interventional neuroradiologist could look like. In addition to traditional assessment of theoretical knowledge practical skills are measured by the use of endovascular simulators yielding objective and quantitative data for the evaluation of the current performance status of each participant as well as the evolution of technical competency over time. This approach also has the scientific potential to explore the interaction between theoretical knowledge, experience, and practical skills and to get transferred into regular training programs.

## Supporting Information

S1 Table Measurement of experience, knowledge, and practical skills. (DOCX)

S2 Table Results of the bivariate and multivariate prediction of skills from experience and knowledge. (DOCX)
