# Supplementary material for: Quantitative Evaluation of Performance in Interventional Neuroradiology: An Integrated Curriculum Featuring Theoretical and Practical Challenges
Source: PLoS One. 2016 Feb 5;11(2):e0148694. doi: 10.1371/journal.pone.0148694 (PMC4743848; doi:10.1371/journal.pone.0148694)
Supplement: S1 Table — (DOCX) [file pone.0148694.s001.docx]

**S1 Table Measurement of experience, knowledge, and practical skills**

| ***Domain name*** | **Component description** | **Component measurement (values with higher domain score)** | **Component weight** |
| --- | --- | --- | --- |
| *EXPERIENCE* |  |  |  |
| *Years experience* | Progress of radiology training | 7-step Likert-type scale (more) | 1/2 |
|  | Years of experience in neuroradiology | 7-step Likert-type scale (more) | 1/2 |
| *Aneurysm treatment experience* | Number of intracranial aneurysms treated as responsible first operator | 6-step Likert-type scale (more) | 2/3 |
|  | Number of intracranial aneurysms procedures assisted | 6-step Likert-type scale (more) | 1/3 |
| *Thrombosis treatment experience* | Number of thrombectomies performed as responsible first operator | 6-step Likert-type scale (more) | 2/3 |
|  | Number of thrombectomies assisted | 6-step Likert-type scale (more) | 1/3 |
| *Flow-Disrupting Devices experience* | Number of intracranial Flow-Disrupting Devices (for example Woven Endobridge Device [WEB], LUNA) procedures attended | 6-step Likert-type scale (more) | 1/1 |
| *KNOWLEDGE* |  |  |  |
| *Anatomy knowledge* | Anatomy and embryology written multiple choice exam | 5 questions (higher number correct) | 2/3 |
|  | Anatomy oral exam | 4-step Likert-type rating (higher) | 1/3 |
| *Materials knowledge* | Materials and techniques written multiple choice exam | 5 questions (higher number correct) | 2/3 |
|  | Materials and techniques oral exam | 4-step Likert-type rating (higher) | 1/3 |
| *Studies knowledge* | Studies written multiple choice exam | 5 questions (higher number correct) | 2/3 |
|  | Studies oral exam | 4-step Likert-type rating (higher) | 1/3 |
| *Treatment knowledge* | AVM and aneurysms written multiple choice exam | 5 questions (higher number correct) | 8/12 |
|  | Pathophysiology oral exam | 4-step Likert-type rating (higher) | 1/12 |
|  | Natural course oral exam | 4-step Likert-type rating (higher) | 1/12 |
|  | Understanding the situation oral exam | 4-step Likert-type rating (higher) | 1/12 |
|  | Complications oral exam | 4-step Likert-type rating (higher) | 1/12 |
| *PRACTICAL SKILLS* |  |  |  |
| *Aneurysm coiling* | Per cent packing | per cent (more) | 4/16 |
|  | Time to final guide catheter position | seconds (less) | 3/16 |
|  | Complications (perforation/hemorrhage) | yes/no (no) | 3/16 |
|  | Fluoroscopy time | seconds (less) | 2/16 |
|  | Correct first coil selection | yes/no (yes) | 2/16 |
|  | Amount of contrast medium left | unit (less) | 1/16 |
|  | Number of coils | number (less) | 1/16 |
| *Thrombectomy* | Time to final guide catheter position | seconds (less) | 3/6 |
|  | Fluoroscopy time | seconds (less) | 2/6 |
|  | Amount of contrast medium left | unit (less) | 1/6 |
| *WEB treatment* | WEB placement correct | yes/no (yes) | 2/3 |
|  | Procedure time | seconds (less) | 1/3 |
