# Supplementary material for: Quantitative Evaluation of Performance in Interventional Neuroradiology: An Integrated Curriculum Featuring Theoretical and Practical Challenges
Source: PLoS One. 2016 Feb 5;11(2):e0148694. doi: 10.1371/journal.pone.0148694 (PMC4743848; doi:10.1371/journal.pone.0148694)
Supplement: S2 Table — (DOCX) [file pone.0148694.s002.docx]

**S2 Table Results of the bivariate and multivariate prediction of skills from experience and knowledge**

|  | **ANEURYSM COILING SKILLS** | | | | | | **THROMBECTOMY SKILLS** | | | | | | **WEB TREATMENT SKILLS** | | | | | |
| --- | --- | --- | --- | --- | --- | --- | --- | --- | --- | --- | --- | --- | --- | --- | --- | --- | --- | --- |
| ***Predictors*** | **r** | **(95% CI)** | **B.full** | **(95% CI)** | **B.back** | **(95% CI)** | **r** | **(95% CI)** | **B.full** | **(95% CI)** | **B.back** | **(95% CI)** | **r** | **(95% CI)** | **B.full** | **(95% CI)** | **B.back** | **(95% CI)** |
| *DEMOGRAPHICS* |  |  |  |  |  |  |  |  |  |  |  |  |  |  |  |  |  |  |
| *Sex^a^* | -0.019 | (-0.440, 0.402) | -0.142 | (-0.821, 0.537) | - | - | -0.150 | (-0.567, 0.266) | 0.064 | (-0.384, 0.513) | - | - | -0.221 | (-0.632, 0.190) | -0.347 | (-0.905, 0.210) | **-0.359** | **(-0.758, 0.040)^+^** |
| *Age^b^* | -0.083 | (-0.503, 0.337) | 0.048 | (-0.523, 0.620) | - | - | **-0.398** | **(-0.784, -0.011)*** | **-0.329** | **(-0.707, 0.048)^+^** | **-0.356** | **(-0.664, -0.049)*** | -0.156 | (-0.572, 0.260) | -0.069 | (-0.905, 0.210) | - | - |
| *EXPERIENCE* |  |  |  |  |  |  |  |  |  |  |  |  |  |  |  |  |  |  |
| *Years^c^* | 0.032 | (-0.389, 0.453) | -0.082 | (-0.612, 0.448) | - | - | -0.228 | (-0.638, 0.183) | -0.189 | (-0.539, 0.161) | - | - | -0.314 | (-0.714, 0.086) | **-0.366** | **(-0.801, 0.069)^+^** | **-0.320** | **(-0.679, 0.040)^+^** |
| *Aneurysm treatment^c^* | 0.225 | (-0.186, 0.635) | -0.077 | (-0.826, 0.671) | - | - | 0.125 | (-0.293, 0.543) | 0.252 | (-0.242, 0.746) | - | - | 0.070 | (-0.350, 0.491) | 0.055 | (-0.559, 0.669) | - | - |
| *Thrombectomy* | 0.103 | (-0.316, 0.522) | 0.027 | (-0.620, 0.673) | - | - | 0.191 | (-0.223, 0.604) | 0.284 | (-0.143, 0.711) | **0.396** | **(0.057, 0.735)*** | **-0.359** | **(-0.752, 0.034)^+^** | -0.347 | (-0.877, 0.184) | - | - |
| *Flow disruption* | 0.296 | (-0.106, 0.699) | 0.224 | (-0.358, 0.806) | - | - | 0.327 | (-0.071, 0.725) | 0.147 | (-0.238, 0.531) | - | - | 0.125 | (-0.293, 0.543) | 0.061 | (-0.417, 0.539) | - | - |
| *KNOWLEDGE* |  |  |  |  |  |  |  |  |  |  |  |  |  |  |  |  |  |  |
| *Anatomy^c^* | 0.098 | (-0.321, 0.517) | 0.274 | (-0.362, 0.911) | - | - | -0.218 | (-0.629, 0.193) | -0.231 | (-0.652, 0.190) | - | - | 0.279 | (-0.126, 0.684) | **0.497** | **(-0.026, 1.020)^+^** | **0.501** | **(0.101, 0.900)*** |
| *Materials^c^* | **0.431** | **(0.050, 0.811)*** | 0.390 | (-0.216, 0.996) | **0.431** | **(0.050, 0.811)*** | **0.491** | **(0.124, 0.858)*** | 0.176 | (-0.224, 0.577) | **0.308** | **(-0.006, 0.622)^+^** | 0.230 | (-0.180, 0.640) | 0.273 | (-0.224, 0.771) | **0.314** | **(-0.054, 0.682)^+^** |
| *Studies^c^* | 0.188 | (-0.226, 0.602) | 0.199 | (-0.401, 0.799) | - | - | 0.000 | (-0.422, 0.421) | 0.047 | (-0.349, 0.444) | - | - | -0.008 | (-0.429, 0.414) | 0.250 | (-0.243, 0.742) | - | - |
| *Treatment^c^* | -0.044 | (-0.465, 0.376) | -0.104 | (-0.702, 0.494) | - | - | **0.358** | **(-0.012, 0.768)^+^** | **0.699** | **(0.274, 1.064)**** | **0.562** | **(0.229, 0.895)**** | 0.092 | (-0.328, 0.511) | -0.215 | (-0.706, 0.276) | - | - |

r=Pearson product-moment correlation coefficient; B.full=standardized regression coefficient in the model containing all predictors; B.back=standardized regression coefficient in the backward variable selection model; CI=confidence interval; ^a^ female (1) vs male (0); ^b^ ≥40 years (1) vs <40 years (0); ^c^ standardized composite scores with higher values indicating more experience/knowledge; ** p<.01; * p<.05; ^+^ p<.10
